# Time-Dependent Growth of Sputtered MoS_2_ Films on ZnO Nanorods for Enhanced NO_2_ Sensing Performance

**DOI:** 10.3390/mi16060659

**Published:** 2025-05-30

**Authors:** Rishi Ranjan Kumar, Shivam Gupta, Aswin kumar Anbalagan, Afzal Khan, Nyan-Hwa Tai, Chih-Hao Lee, Heh-Nan Lin

**Affiliations:** 1 School of Basic and Applied Sciences, K.R. Mangalam University, Gurugram 122103, Haryana, India; 2 Department of Materials Science and Engineering, National Tsing Hua University, Hsinchu 300044, Taiwan; shivam19gupta@gmail.com (S.G.); nhtai@mx.nthu.edu.tw (N.-H.T.); hnlin@mx.nthu.edu.tw (H.-N.L.); 3 Department of Engineering and System Science, National Tsing Hua University, Hsinchu 300044, Taiwan; aswinakumar89@gmail.com (A.k.A.); chlee@mx.nthu.edu.tw (C.-H.L.); 4 State Key Laboratory of Fluid Power and Mechatronic Systems, Zhejiang Provincial Key Laboratory for Atomic-Level Manufacturing, School of Mechanical Engineering, Zhejiang University, Hangzhou 310058, China

**Keywords:** sputtering, MoS_2_/ZnO nanostructures, NO_2_ gas sensing, synergistic effect

## Abstract

Molybdenum disulfide (MoS_2_) has gained attention for its promising gas-sensing capabilities due to its high surface area and tunable electronic properties. In this study, we investigate the time-dependent growth (under constant conditions) of sputtered MoS_2_ films on ZnO nanorods and their impact on NO_2_ sensing performance. ZnO nanorods, synthesized via a hydrothermal method, provide a high-surface-area template to enhance charge transport and gas adsorption. Gas-sensing experiments revealed a strong correlation between MoS_2_ thickness and NO_2_ response, with the 25-min-sputtered MoS_2_ film exhibiting the highest response of 20.9%. The synergistic interaction between MoS_2_ and ZnO nanorods facilitated charge transfer and enhanced adsorption sites for NO_2_ molecules. These findings emphasize the critical role of time-dependent growth of MoS_2_ film in modulating gas-sensing performance and provide insights into designing high-sensitivity NO_2_ sensors at room temperature. This study contributes to the development of hybrid MoS_2_/ZnO nanostructures for next-generation environmental monitoring applications.

## 1. Introduction

Air pollution has become a significant environmental and public health concern due to the increasing release of hazardous gases from industrial, vehicular, and agricultural activities [1]. Among these pollutants, nitrogen dioxide (NO_2_) is a highly toxic gas known for its adverse effects on human health and the environment [2]. Exposure to even low concentrations of NO_2_ can cause respiratory disorders, cardiovascular diseases, and contribute to the formation of acid rain and photochemical smog [3]. Major sources of NO_2_ emissions include automobile exhausts, industrial combustion processes, and agricultural waste decomposition [4]. Sensors play a vital role in detecting chemical, biological, and environmental changes across diverse applications [5]. Given its harmful impact, the development of reliable, highly sensitive, and selective gas sensors for NO_2_ detection is crucial for real-time environmental monitoring and pollution control [6]. In this regard, semiconductor-based gas sensors have gained significant attention due to their low cost, high sensitivity, and ease of fabrication [7]. Over the past two decades, semiconductor metal–oxide nanomaterials (SMONs) such as ZnO [8,9], SnO_2_ [10], TiO_2_ [11], WO_3_ [12], and In_2_O_3_ [13] have been widely explored as chemoresistive gas sensors due to their high sensitivity, fast response, low power consumption, cost-effective synthesis, and miniaturization potential. Among them, ZnO-based sensors have gained significant attention [14,15].

Transition-metal dichalcogenide (TMD)-based materials have emerged as promising candidates for a wide range of applications, including transistors, gas sensors, wearable electronics, energy storage, and catalysis [16,17,18]. TMDs possess a thickness-dependent bandgap and exceptional thermal stability, which enhance charge transport and gas adsorption properties, making them ideal candidates for next-generation sensing technologies. Among various TMDs, molybdenum disulfide (MoS_2_) has been extensively investigated for gas-sensing applications in various forms, including monolayer and multilayer nanosheets, liquid-phase exfoliated sensors on flexible substrates, and atomic layer deposition-based sensors [19,20]. Since gas sensing primarily occurs at the material’s surface, various surface functionalization strategies such as doping, noble metal catalyst loading, metal oxide incorporation, and nanocomposite formation have been established as effective approaches to enhance sensing performance [21,22,23]. Among these, surface doping plays a crucial role as it not only influences the concentration of free charge carriers but also regulates their mobility. First-principles studies indicate that transition metal doping can significantly modify the electronic structure and magnetic properties while also improving gas molecule adsorption and activation, leading to enhanced sensor performance [24]. More recently, MoS_2_ deposited via direct current sputtering has been utilized as a NO_2_ sensor, where a transition from the as-deposited state to the oxidized state resulted in a decrease in conductance. Neetika et al. investigated a MoS_2_ (n-type)/Si (p-type) heterojunction-based NO_2_ gas sensor, reporting a response of less than 32% at 100 ppm NO_2_ when operated at 150 °C [25]. However, by optimizing the operating temperature, the sensor’s performance could be further enhanced. It is well-established that p–n or n–p heterojunction-based gas sensors are highly attractive due to their enhanced gas sensing performance, which arises from significant modulation in current across the junction [26].

In this study, MoS_2_/ZnO heterojunction nanorods were grown using the DC magnetron sputtering technique. The structural properties, surface morphology, and room-temperature NO_2_ gas-sensing properties of the as-grown ZnO nanorods and MoS_2_/ZnO heterojunction nanorods were systematically analyzed. The MoS_2_/ZnO heterostructure nanorods exhibited excellent gas-sensing performance, demonstrating their potential for the fabrication of high-efficiency room-temperature NO_2_ gas sensors.

## 2. Materials and Methods

### 2.1. Synthesis of ZnO Nanorods, MoS_2_ Films, and MoS_2_/ZnO Heterostructures

**Scheme 1:**
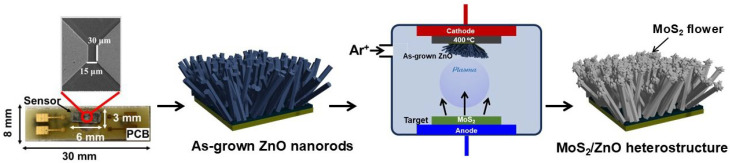
Schematic representation for the growth of MoS_2_/ZnO heterostructures.

The electrodes were fabricated on a thermally oxidized silicon wafer using standard photolithography, electron-beam (e-beam) evaporation, and lift-off techniques. The interdigitated two-finger electrodes consist of a Ti (15 nm)/Au (300 nm) bilayer. The channel gap between the electrodes is 15 µm, which defines the active sensing region where the MoS_2_/ZnO composite was deposited. The growth of ZnO nanorods was realized by a hydrothermal method in our previous work [15]. The MoS_2_ target (2” diameter) with high purity (99.99%) was purchased from Ultimate Materials Technology Co., Ltd., (UMAT), Hsinchu, Taiwan. The base pressure of the sputtering chamber was maintained at approximately 1 × 10^−6^ Torr. Sputtering was performed using a DC power of 20 W in an argon atmosphere at a working pressure of 5 × 10^−3^ Torr [27]. MoS_2_ films were deposited at a substrate temperature of 400 °C with an Ar flow rate of 40 sccm. The film thickness was controlled by varying the deposition time to 2.5, 5, and 25 min, respectively. The resulting samples were labeled M1Z, M2Z, and M3Z, corresponding to the increasing deposition durations. The approximate deposition rate for our MoS_2_ film was around 2 nm/min. The schematic representations of the synthesis of MoS_2_/ZnO heterostructures are shown in Scheme 1.

### 2.2. Gas-Sensing Measurements

A 500 ppm NO_2_ gas diluted with N_2_ gas at a flow rate of 8 sccm was introduced into an 8 L chamber. The electrical measurements were obtained at an ambient pressure of 1 atm. All sensors operated at room temperature (27 °C). The sensor chip was mounted within a sensing module that included a 365 nm UV LED for activation. The relative humidity (RH) during gas sensing was maintained at 55% except for the humidity effect tests. For low RH (25%), dry air was introduced into the chamber and monitored using a humidity sensor. For high RH (85%), a homemade humidifier generated water vapor to achieve the desired level. The sensor current was measured at a 5 V bias using a source-measure unit (Keithley 2400). The sensing response *A* was calculated from the following equation.

$$A(\%)=\frac{I_{g}-I_{a}}{I_{a}}\times 100\%$$

where *I_g_* and *I_a_* are the sensor currents in NO_2_ gas and air, respectively.

### 2.3. Characterization

The surface morphologies of the MoS_2_, ZnO, and MoS_2_/ZnO bilayer thin films were examined under a field-emission scanning electron microscope (SEM, JEOL JSM-6500F, Tokyo, Japan). Energy-dispersive X-ray spectroscopy (EDS, Hitachi S-4800, Tokyo, Japan) was used to determine the elemental composition and mapping analysis. The chemical state information of the MoS_2_/ZnO heterojunction thin-film sample was determined by X-ray photoelectron spectroscopy (XPS, Ulvac-PHI 5000 Versa Probe II, Chigasaki, Japan). Photoluminescence (PL) spectra were recorded using a Hitachi F-7000 (Tokyo, Japan) fluorescence spectrophotometer equipped with a 150 W Xe lamp and an excitation wavelength of 325 nm.

## 3. Results

### 3.1. Structural Properties

**Figure 1:**
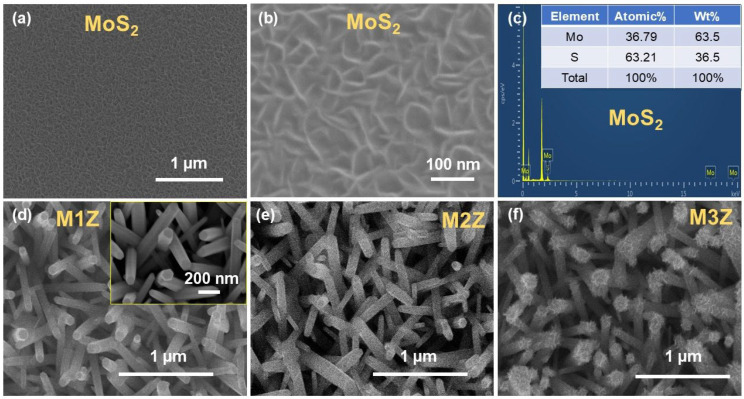
FESEM images of (**a**,**b**) MoS_2_ thin films and (**c**) the EDX spectrum of a MoS_2_ thin film. FESEM images of MoS_2_/ZnO heterostructures for samples (**d**) M1Z, (**e**) M2Z, and (**f**) M3Z, respectively.

Figure 1a,b show the FE-SEM images of the MoS_2_ film grown by the sputtering method. The SEM image reveals a uniform and compact surface morphology with densely packed nanograins. The film exhibits a continuous structure without visible cracks, indicating good adhesion and coverage on the underlying substrate. The EDS spectrum (Figure 1c) confirms the presence of molybdenum (Mo) and sulfur (S) elements, indicating the successful deposition of MoS_2_ on the Si substrate. The ZnO nanorods, with an average diameter of approximately 100 nm, were uniformly distributed across the surface, as shown in the inset of Figure 1d. As the growth time increases, the flower-like morphology of MoS_2_ becomes more pronounced, with larger and more well-defined nanosheets forming due to the continued accumulation and crystallization of Mo and S atoms on the ZnO nanorod surface. Figure 1d presents a top-view SEM image of the ZnO nanorods, revealing their predominantly vertical growth with random orientations. As the MoS_2_ sputtering time increases, the surface of the nanorods becomes progressively rougher (Figure 1e), indicating the gradual deposition and coverage of MoS_2_ on the ZnO surface. The SEM image of the MoS_2_/ZnO heterostructure reveals a distinct flower-like morphology (shown in Figure 1f), where the MoS_2_ nanosheets are elegantly grown on the tips and sidewalls of vertically aligned ZnO nanorods. This petal-like arrangement of MoS_2_ gives the overall structure a floral appearance, enhancing the surface area and providing a high density of active sites. The growth of MoS_2_ on the tips of the ZnO nanorods occurs because the tips possess higher surface energy, increased density of dangling bonds, and often more oxygen vacancies compared to the sidewalls, making them energetically favorable sites for nucleation. During sputtering, the adatoms (Mo and S) tend to migrate and accumulate at these reactive tip sites, leading to the vertical growth of MoS_2_ nanosheets and the formation of a flower-like morphology.

**Figure 2:**
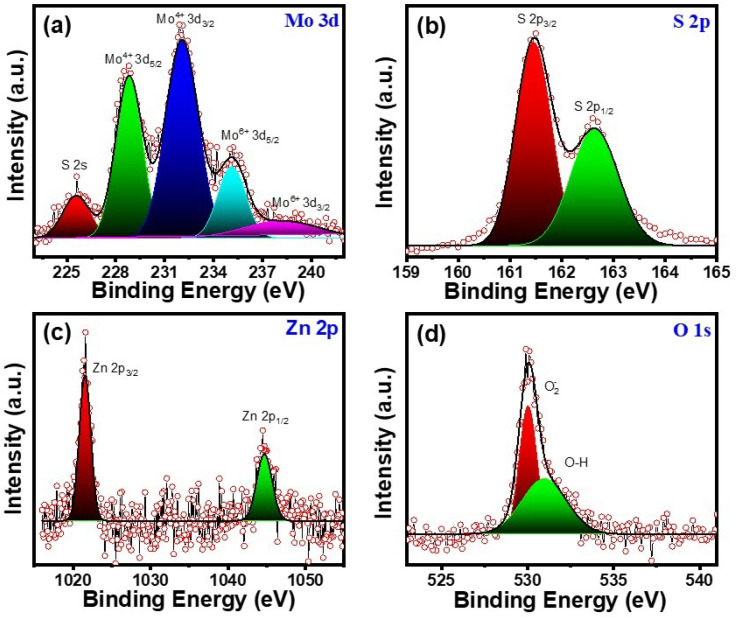
High-resolution XPS spectra of M3Z for (**a**) Mo 3d, (**b**) S 2p, (**c**) Zn 2p, and (**d**) O1s, respectively.

The high-resolution XPS analysis of the MoS_2_/ZnO heterostructure sputtered for 25 min (M3Z) provides detailed insights into the elemental composition and chemical states of Mo, S, Zn, and O, as shown in Figure 2. The Mo 3d spectrum exhibits two prominent peaks at approximately 228.8 eV and 232 eV, corresponding to Mo 3d_5/2_ and Mo 3d_3/2_, respectively, confirming the presence of Mo in the +4 oxidation state. Additionally, two distinct peaks at 235.1 eV and 238.5 eV are observed, corresponding to the Mo^6+^ 3d_5/2_ and Mo^6+^ 3d_3/2_ states, respectively. These higher oxidation states are attributed to the presence of geometric edge defects (Figure 2a). The peak at the binding energy of 225.5 eV is primarily ascribed to S 2s. The S 2p spectrum shows peaks around 162.2 eV and 163.4 eV, attributed to S 2p_3/2_ and S 2p_1/2_, respectively, further validating the formation of MoS_2_ (Figure 2b). The Zn 2p spectrum reveals peaks at ~1021.6 eV and ~1044.7 eV, corresponding to Zn 2p_3/2_ and Zn 2p_1/2_, indicating the presence of Zn^2+^ in ZnO [28]. Additionally, the O 1s spectrum displays a peak near 530.1 eV, associated with lattice oxygen in ZnO, along with a shoulder at higher binding energy, which may be attributed to surface-adsorbed species or oxygen-related defects. No other impurity was observed on the surface of the sample other than Mo, S, Zn, and O elements. These results collectively confirm the successful deposition of MoS_2_ onto ZnO nanorods while maintaining the integrity of both components in the heterostructure.

**Figure 3:**
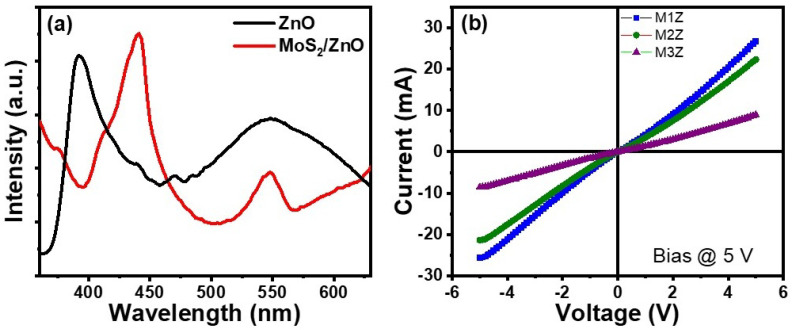
(**a**) PL spectrum of as-grown ZnO nanorods and MoS_2_/ZnO heterostructure. (**b**) *I–V* characteristics for M1Z, M2Z, and M3Z, respectively.

Photoluminescence (PL) analysis reveals significant changes in the emission properties upon the formation of the MoS_2_/ZnO heterostructure (Figure 3a). For pristine ZnO nanorods, a strong near-band-edge (NBE) emission peak is observed at 392 nm, along with a broad visible emission attributed to oxygen vacancies. However, in the MoS_2_/ZnO heterostructure, the main emission peak shifts to 441 nm, indicating a strong interfacial interaction and possible charge transfer between MoS_2_ and ZnO. Additionally, the oxygen vacancy-related emission becomes narrower and less intense, suggesting a reduction in surface defects due to MoS_2_ coverage and improved passivation at the interface.

The current–voltage (*I–V*) characteristics of the MoS_2_/ZnO heterostructures with varying MoS_2_ sputtering times (M1Z, M2Z, and M3Z) were recorded under a bias voltage range of −5 V to +5 V (Figure 3b). All samples exhibit a linear *I–V* relationship, indicating ohmic contact behavior. However, the current decreases progressively with increasing sputtering time, with M1Z showing the highest current and M3Z the lowest. This decline in current can be attributed to the increasing thickness of the MoS_2_ layer, which may act as a barrier to charge transport by increasing resistance and limiting carrier mobility across the heterojunction. The denser MoS_2_ coverage at longer deposition times could also reduce the number of active charge transfer pathways between MoS_2_ and ZnO.

### 3.2. Gas-Sensing Characteristics

**Figure 4:**
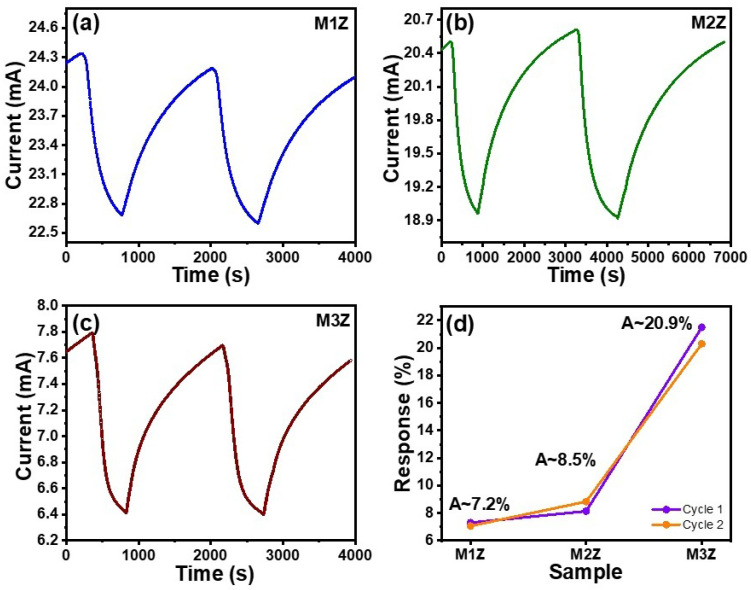
NO_2_ gas-sensing response of MoS_2_/ZnO heterostructures (**a**) M1Z, (**b**) M2Z, (**c**) M3Z. (**d**) Comparison of responses at 500 ppb.

The NO_2_ gas-sensing performance of the MoS_2_/ZnO heterostructure samples (M1Z, M2Z, and M3Z) was evaluated at a concentration of 500 ppb, and the results demonstrate a significant enhancement in the response with increasing MoS_2_ deposition time. Among the three samples, M1Z (2.5-min sputtering) exhibited an average response of 7.2% (Figure 4a), while M2Z (5-min sputtering) showed a slightly higher response of 8.5% (Figure 4b), indicating the initial improvement in sensing performance with increased MoS_2_ coverage. Notably, the M3Z sample (25-min sputtering) displayed a substantial enhancement, achieving an average response of 20.9% (Figure 4c). This marked increase can be attributed to the well-developed flower-like MoS_2_ structure on the ZnO nanorods, which provides a larger surface area, more active sites, and improved charge separation, thereby facilitating more efficient adsorption and reaction with NO_2_ molecules. The hybrid heterostructure benefits from the synergistic effect between MoS_2_ and ZnO, where MoS_2_ acts as a p-type semiconductor and ZnO as an n-type, forming a p–n junction that enhances the gas-sensing response by promoting electron depletion in ZnO upon NO_2_ exposure. Furthermore, all samples demonstrated complete recovery after gas removal, highlighting their excellent reversibility and stability. A comparative response curve plotted over two cycles, as shown in Figure 4d, clearly illustrates the increasing trend in average response from M1Z to M3Z, validating the role of MoS_2_ thickness and morphology in tuning the gas-sensing behavior of the MoS_2_/ZnO heterostructures.

**Figure 5:**
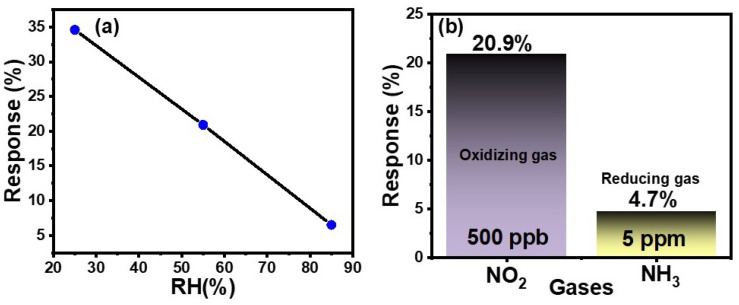
(**a**) Gas-sensing response of the M3Z sensor under varying relative humidity conditions (25%, 55%, and 85%). (**b**) Selectivity analysis toward NO_2_ (500 ppb) and NH_3_ (5 ppm).

Humidity tests were conducted for the M3Z sample at relative humidity (RH) levels of 25%, 55%, and 85%, resulting in NO_2_ sensing responses of 34.6%, 20.9%, and 6.5%, respectively (Figure 5a). The observed decline in response to increasing humidity is attributed to the competitive adsorption of water molecules on the active sensing sites, which interferes with the adsorption and reaction of NO_2_ molecules on the MoS_2_/ZnO surface. Additionally, a selectivity test was performed using two representative gases, NO_2_ (an oxidizing gas) and NH_3_ (a reducing gas), at concentrations of 500 ppb and 5 ppm, respectively (Figure 5b). The sensor exhibited a significantly higher response to NO_2_ (20.9%) compared to NH_3_ (4.7%), highlighting its strong selectivity toward oxidizing gases. This enhanced selectivity can be attributed to the favorable charge transfer interactions between the electron-withdrawing NO_2_ and the p-type MoS_2_ in the MoS_2_/ZnO heterostructure, which leads to a more pronounced resistance change compared to the interaction with the electron-donating NH_3_.

Various MoS_2_/ZnO composite structures have been developed using different synthesis methods, as outlined in Table 1. Techniques such as hydrothermal growth, solvothermal methods, sputtering, and chemical vapor deposition (CVD) have been employed to tailor the morphology and interface of the heterostructures. Each method influences the structural and electronic properties, which directly impact device performance. Notably, sputtered MoS_2_ films on ZnO nanorods offer uniform coating and controlled thickness, making them suitable for sensing applications. These diverse fabrication strategies highlight the versatility of MoS_2_/ZnO composites for functional device development.

### 3.3. Gas-Sensing Mechanism

**Figure 6:**
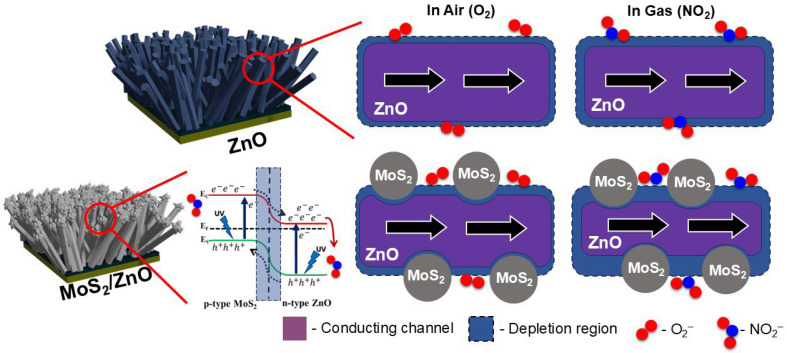
Schematic illustration of depletion layer expansion upon gas adsorption and energy band alignment at the p–n heterojunction.

The gas-sensing mechanism of sputtered MoS_2_/ZnO heterostructures toward NO_2_ primarily relies on the modulation of charge carrier concentration at the interface of the p–n heterojunction formed between p-type MoS_2_ and n-type ZnO (Figure 6). When these two materials are combined, electrons from ZnO diffuse into MoS_2_ and holes from MoS_2_ diffuse into ZnO until their Fermi levels align, leading to the formation of a depletion region at the interface. This built-in electric field facilitates charge separation and enhances the sensitivity of the heterostructure. In air, oxygen molecules adsorb on the surface of the heterostructure and capture free electrons from ZnO, forming ionized oxygen species (O_2_^−^), which further widen the depletion layer and decrease conductivity.

$$O_{2}+e^{-}\to O_{2}^{-}\left(\mathrm{ads}\right)$$

Upon exposure to NO_2_ gas, which is a strong oxidizing agent, additional electrons from the conduction band of ZnO and MoS_2_ are withdrawn due to the high electron affinity of NO_2_. This further increases the depletion width and significantly reduces the overall conductivity of the sensor.

$${\mathrm{NO}}_{2}+e^{-}\to {\mathrm{NO}}_{2}^{-}\left(\mathrm{ads}\right)$$

When NO_2_ is pumped out from the system, the desorption of chemisorbed NO_2_ molecules occurs slowly under ambient conditions. However, under UV activation, the presence of photogenerated holes facilitates and accelerates the desorption process.

$${\mathrm{NO}}_{2}^{-}+h^{+}\to {\mathrm{NO}}_{2}$$

In the MoS_2_/ZnO system, the flower-like morphology of MoS_2_ provides a high surface area and abundant active sites for gas adsorption, while the underlying ZnO nanorods offer rapid electron transport pathways. The synergistic effect between MoS_2_ and ZnO results in enhanced gas-sensing performance, including higher sensitivity and faster response/recovery. After the removal of NO_2_, the adsorbed species desorb from the surface, restoring the original electrical state, thus enabling full recovery of the sensor signal.

## 4. Conclusions

In this study, MoS_2_/ZnO heterostructures were successfully fabricated using a DC sputtering technique with varying MoS_2_ deposition durations. Structural and morphological analyses confirmed the uniform growth of flower-like MoS_2_ structures on vertically aligned ZnO nanorods, which enhanced the surface area and provided abundant active sites for gas interaction. XPS analysis verified the chemical states of Mo, S, Zn, and O, confirming the successful formation of the heterostructure. Photoluminescence measurements revealed a redshift in emission and a narrowing of oxygen vacancy-related peaks upon MoS_2_ decoration, indicating strong interfacial interactions. Notably, NO_2_ gas-sensing performance at 500 ppb significantly improved with longer sputtering times, with the M3Z sample showing the highest average response of 20.9% and complete recovery. The enhanced sensing behavior is attributed to the synergistic effect of the p–n heterojunction and the increased surface reactivity of the MoS_2_ nanoflowers. These findings demonstrate the potential of sputtered MoS_2_/ZnO heterostructures for high-performance, low-concentration NO_2_ gas-sensing applications.

## Figures and Tables

**Table 1:** Summary of MoS_2_/ZnO composite structures prepared by different synthesis methods and their key features.

Material	Nanocomposite Synthesis Method		Temperature	Concentration	Response (%)	Ref.
	MoS_2_	ZnO				
MoS_2_/Cu_x_O/ZnO	Photo deposition	Hydrothermal method	RT	500 ppb	890	[29]
MoS_2_/ZnO nanowires	Mo—Sputtering S—Sulfurization	Hydrothermal method	200 °C	50 ppm	31.2	[30]
MoS_2_/ZnO nanorods	Hydrothermal method	Hydrothermal method	RT	50 ppm	34.91	[31]
MoS_2_/ZnO composites	Hydrothermal method	Hydrothermal method	200 °C	10 ppm	33.77	[32]
MoS_2_/ZnO	Liquid exfoliation	Hydrothermal method	RT	200 ppb	188	[33]
MoS_2_/ZnO	MoS_2_ as target—Sputtering	Hydrothermal method	RT	500 ppb	20.9	This work

## Data Availability

The data that support the findings of this study are available from the corresponding author upon reasonable request.
